# A Versatile and Scalable Platform That Streamlines Data Collection for Patient-Centered Studies: Usability and Feasibility Study

**DOI:** 10.2196/38579

**Published:** 2022-09-14

**Authors:** Haley Huang, Sofia Aschettino, Nasim Lari, Ting-Hsuan Lee, Sarah Stothers Rosenberg, Xinyi Ng, Stella Muthuri, Anirudh Bakshi, Korrin Bishop, Hussein Ezzeldin

**Affiliations:** 1 IBM Consulting IBM Bethesda, MD United States; 2 Center for Biologics Evaluation and Research United States Food and Drug Administration Silver Spring, MD United States; 3 Gevity Consulting Inc. Accenture Ottawa, ON Canada; 4 Korrin Bishop Writing & Editing Kodak, TN United States

**Keywords:** mobile app, patient experience data, data-collection app, mobile phone, usability, mHealth app, feasibility, user centered, eHealth, patient-generated data

## Abstract

**Background:**

The Food and Drug Administration Center for Biologics Evaluation and Research (CBER) established the Biologics Effectiveness and Safety (BEST) Initiative with several objectives, including the expansion and enhancement of CBER’s access to fit-for-purpose data sources, analytics, tools, and infrastructures to improve the understanding of patient experiences with conditions related to CBER-regulated products. Owing to existing challenges in data collection, especially for rare disease research, CBER recognized the need for a comprehensive platform where study coordinators can engage with study participants and design and deploy studies while patients or caregivers could enroll, consent, and securely participate as well.

**Objective:**

This study aimed to increase awareness and describe the design, development, and novelty of the Survey of Health and Patient Experience (SHAPE) platform, its functionality and application, quality improvement efforts, open-source availability, and plans for enhancement.

**Methods:**

SHAPE is hosted in a Google Cloud environment and comprises 3 parts: the administrator application, participant app, and application programming interface. The administrator can build a study comprising a set of questionnaires and self-report entries through the app. Once the study is deployed, the participant can access the app, consent to the study, and complete its components. To build SHAPE to be scalable and flexible, we leveraged the open-source software development kit, Ionic Framework. This enabled the building and deploying of apps across platforms, including iOS, Android, and progressive web applications, from a single codebase by using standardized web technologies. SHAPE has been integrated with a leading Health Level 7 (HL7®) Fast Healthcare Interoperability Resources (FHIR®) application programming interface platform, 1upHealth, which allows participants to consent to 1-time data pull of their electronic health records. We used an agile-based process that engaged multiple stakeholders in SHAPE’s design and development.

**Results:**

SHAPE allows study coordinators to plan, develop, and deploy questionnaires to obtain important end points directly from patients or caregivers. Electronic health record integration enables access to patient health records, which can validate and enhance the accuracy of data-capture methods. The administrator can then download the study data into HL7® FHIR®–formatted JSON files. In this paper, we illustrate how study coordinators can use SHAPE to design patient-centered studies. We demonstrate its broad applicability through a hypothetical type 1 diabetes cohort study and an ongoing pilot study on metachromatic leukodystrophy to implement best practices for designing a regulatory-grade natural history study for rare diseases.

**Conclusions:**

SHAPE is an intuitive and comprehensive data-collection tool for a variety of clinical studies. Further customization of this versatile and scalable platform allows for multiple use cases. SHAPE can capture patient perspectives and clinical data, thereby providing regulators, clinicians, researchers, and patient advocacy organizations with data to inform drug development and improve patient outcomes.

## Introduction

### Barriers to the Study of Rare Diseases

Treatments for rare diseases comprise the fastest-growing subcategory of novel drugs and biologics approvals in the United States [1]. In 2020, 58% of all novel drugs approved by the US Food and Drug Administration (FDA) and 20% of all novel biological products were designated to treat rare diseases [2]. In the United States, a rare disease, as defined by the Orphan Drug Act of 1983, is a condition that affects <200,000 people in the United States [3]. However, despite significant progress in rare disease therapeutic development, *only a few hundred of the 7000 known rare diseases currently have an FDA-approved treatment* [4]. This leaves many patients with rare diseases with few or no treatment options.

Research and development for the treatment of rare diseases involve several challenges, including underdiagnosis and delayed diagnosis [5,6]. In addition, there is insufficient knowledge of the natural history of most rare diseases. Related to this is the heterogeneous pathogenesis of many rare diseases. Not all rare diseases present or progress in the same way from individual to individual, which complicates drug development [1].

The lack of information on the natural history of many rare diseases stems from the overall low prevalence of the diseases, making it difficult to recruit and retain sufficient population sizes for natural history studies and clinical trials. This includes gathering appropriate control groups against which to compare health outcomes in patients with rare diseases [7].

Furthermore, given the small population sizes available for these studies, other constraints arise. Patients are often widely dispersed geographically, which creates logistical barriers. Patients may have limited access to clinical sites where trials are conducted, which can increase the burden for those participating in the trials [8,9].

Owing to these challenges, clinical evidence on rare diseases is often limited because of a lack of sufficient and robust clinical data [10]. This negatively affects the quality of scientific evidence required to support clinical and regulatory decision-making.

An intuitive, comprehensive platform, such as the one described in this paper, presents an opportunity to confront these barriers and strengthen the clinical evidence base by making research on rare diseases more feasible. Specifically, it may be able to reduce the high burdens of travel, time, and overall fatigue faced by patients and their caregivers when participating in rare disease studies at remote clinical sites. The Survey of Health and Patient Experience (SHAPE) participant app can easily collect patient experience data regardless of their location or time. Thus, the use of a mobile app could accelerate meeting the unmet needs of patients with rare diseases by helping researchers gain greater knowledge on disease conditions.

### Literature Review of Data-Collection Approaches for Rare Diseases

In recent years, innovative approaches have been developed offering substantial promise in promoting more efficient and effective ways of collecting data for epidemiological and clinical studies. These approaches can be applied to overcome methodological and logistical challenges inherent to rare disease research. A literature review was conducted to assess existing mobile apps and tools available for the collection of self-reported patient data used in clinical studies. A targeted search was conducted in PubMed by using the keywords *patient reported outcomes* and *patient reported outcome measures*. Additional details on this search strategy are included in Multimedia Appendix 1.

The search identified numerous tools available for individual diseases or outcomes under study [11-20], as well as remappable resources (ie, migrating data from one system to another), such as REDCap (Research Electronic Data Capture; Vanderbilt University) and the FDA MyStudies app [21,22]. Most single-study apps are limited to the disease or condition under investigation, such as Parkinson disease and asthma [11,12]. The FDA MyStudies app collects information that is unavailable in their electronic health records (EHRs) directly from patients and combines it with the data from their EHRs [22]. REDCap is a web-based application used to create surveys and research forms to capture data for clinical research [21]. These tools were assessed to understand the current state of the field regarding the existing functionality, applications, strengths, and gaps.

### Purpose of the SHAPE Platform

The FDA Center for Biologics Evaluation and Research (CBER) Biologics Effectiveness and Safety (BEST) Initiative recognizes the need for innovative approaches that facilitate efficient and effective ways of collecting fit-for-purpose data for patient-centered studies. To respond to this need, the Biologics Effectiveness and Safety Initiative sought to design and develop the SHAPE platform.

SHAPE was developed to provide an open-source platform that study coordinators and research teams can use to customize studies and scale data-collection efforts. It was designed with interoperability in mind to allow seamless health data exchange and integration with EHR systems while meeting the FDA’s regulatory requirements regarding data security and traceability. SHAPE also facilitates the direct data capture of patient health experiences with a range of health conditions, symptoms, and health care services, as well as the collection of end points that may be relevant to regulators, clinicians, researchers, and patient advocacy organizations. These capabilities allow the collection of longitudinal data outside traditional onsite methods while reducing the burden that patients and their caregivers may face when participating in conventional studies at remote clinical sites, such as travel, costs, time, and overall fatigue. SHAPE is intuitive and highly customizable and offers a wide range of functionalities (eg, questionnaires, self-reports, and EHR linkage), providing a cost-effective solution for organizations to host multiple studies with little additional cost while maintaining control of the platform. SHAPE’s codebase is shared on the FDA public GitHub and is available to any organization interested in leveraging the codebase to set up their own instance of SHAPE [23].

The objective of this study was to describe the design, development, and novelty of SHAPE; its functionality and applications; quality improvement efforts; and plans for its enhancement.

## Methods

### Overview

**Figure 1:**
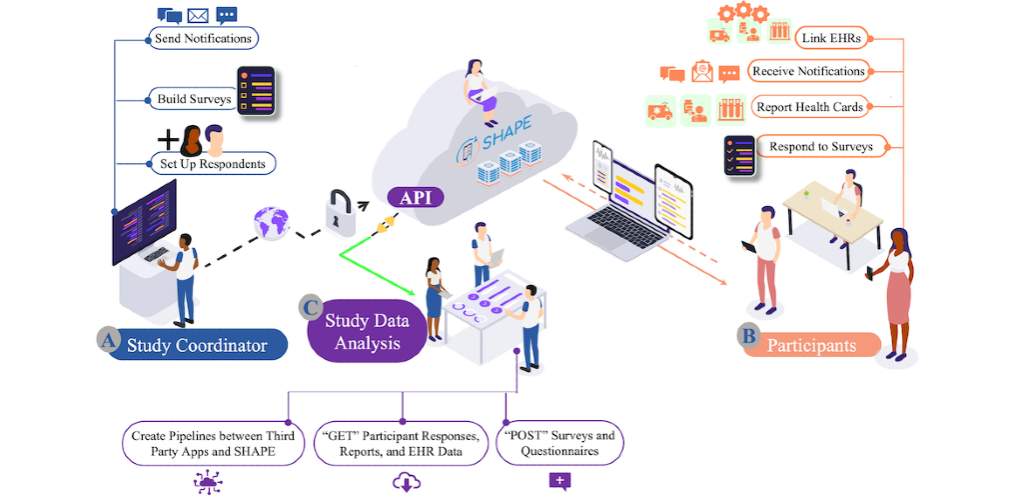
SHAPE overall architecture. API: application programming interface; EHR: electronic health record; SHAPE: Survey of Health and Patient Experience.

SHAPE is a user-friendly platform that teams can leverage to customize studies and scale data-collection efforts to advance health research. It comprises the following three parts:

1. *Administrator application* that study coordinators and administrators can use to develop and deploy study questionnaires and share important information and resources with participants, shown as *A* in Figure 1
2. *Participant app* that allows research participants to explicitly consent to and participate in patient-centered studies through their mobile device, shown as *B* in Figure 1
3. *Application programming interface* (API) that study coordinators can use to push and pull data to SHAPE, shown as *C* in Figure 1

Figure 1 shows the overall SHAPE architecture, highlighting major features and displaying the various SHAPE users and how they interact with the platform.

### Definitions

Throughout this paper, the following terms refer to the corresponding elements of the SHAPE platform:

1. *SHAPE* refers to the platform as a whole.
2. *SHAPE administrator application* refers to the application component used by study coordinators and other administrators.
3. *SHAPE participant app* refers to the app component used by patients and caregivers participating in a SHAPE-based study.
4. *SHAPE API* refers to the API study coordinators can use.

In addition, we use the following terms to further describe the platform and explain the different use scenarios:

1. *Participant* refers to the individual completing a questionnaire in the SHAPE participant app. This can be either a patient or a caregiver who is responding on the patient’s behalf. For instances where it is important to make a distinction, we use *patient* or *caregiver* to identify the respondent specifically.
2. *Study* refers to the questionnaires and health events developed with SHAPE to research a specific question about patient experiences. Although SHAPE uses the term *survey* within the platform, we have chosen to use the term *study* throughout this paper to clarify that research teams can use SHAPE not just for surveys but also for developing comprehensive studies, including longitudinal studies that may use multiple questionnaires over time.
3. *Questionnaire* refers to the individual sets of questions that study administrators can create within SHAPE for participants to answer.

### Design and Development

#### Agile Application Development Process

The SHAPE team comprised members from the FDA CBER, IBM, and National Organization for Rare Disorders (NORD) and patient advocates and caregivers connected to NORD. This team used an agile-based software development process, using a combination of principles, methodologies, and delivery expertise. The team worked closely with other subject matter experts to gain a deep understanding of the outcome vision for SHAPE, as well as the requirements and organizational processes needed.

Using an agile-based approach for developing SHAPE had many benefits. An emphasis on stakeholder engagement allowed the team to work with regulators, caregivers, study coordinators, and clinicians to understand and address their current challenges. This included greater insights into the limitations of current methods for recruiting and interacting with patients. The team was able to use stakeholder knowledge to develop features and designs to meet their needs.

The SHAPE team designed, developed, and implemented functionality in 2-week cycles, completing >60 sprints to date.

#### SHAPE Architecture

To build SHAPE as a scalable and flexible platform, the team used an open-source software development kit—Ionic Framework [24]—to build the various SHAPE components and leverage its easy deployment functionality. This allowed the team to deploy SHAPE across multiple operating systems, including iOS, Android, and progressive web applications from a single codebase, using standardized web technologies.

SHAPE is hosted in a Google Cloud environment. This cloud environment provides SHAPE with a secure infrastructure that is scalable while requiring minimal associated costs. These features allow support for large user base populations.

The SHAPE participant app is available in both the Apple App Store and Google Play Store for user downloads. Most updates to the SHAPE participant app are automatically pushed to the users’ mobile devices without the need for a user-initiated upgrade through the app stores. If the development team implements a major feature that is considered a binary update, app users must update the SHAPE participant app via their operating system’s app store.

Currently, studies in SHAPE are by invitation only. Study administrations provide participants with an ID to be used during registration through the SHAPE participant app. To enroll participants in any study, the administrator has two options:

1. Batch input participants by uploading a CSV file containing unique, non–personally identifiable codes
2. Manually enter this information by using a participant import wizard within the SHAPE administrator application

The SHAPE administrator application enables study administrators to build a study, upload participant study cohorts, and communicate with the cohorts through 1 interface. The study built on the SHAPE administrator app is then deployed to participants through the SHAPE participant app.

Each study comprises a set of questionnaires and self-reports. Self-reports are a set number of predetermined questions—curated by study administrators—that are always accessible to participants for ad hoc data submission. These can be health event questions on the occurrence of a medical diagnosis (eg, infection), symptom (eg, fatigue), or medical visit (eg, emergency room or primary care visit). Participants can report these at any point during the time frame of the study by using the SHAPE participant app.

SHAPE is secure and restricted to only administrators and participants of official studies leveraging SHAPE for data-collection efforts. As a result, the apps require authentic log-in credentials to gain access. To ensure a secure connection, the team has developed the SHAPE API, which allows study administrators with approved credentials to automatically push study-specific data to the SHAPE administrator application and pull collected participant data by developing their own code that hits the API target end point. Partner platforms can set up a seamless integration connection if the user is authenticated to connect to the end points.

SHAPE’s flexible and scalable solution architecture allows for administrator multitenancy. This restricts access for accounts based on their organization, enabling users to only view and export their organization’s studies and participant data. All organizations must work with the SHAPE team to request an administrator account, which will create their own organization-specific sandbox. A benefit of the multitenancy feature is that it allows for multiple groups within an organization to leverage SHAPE without requiring the development and maintenance of their own instance of SHAPE. The cost and time savings are substantial, as the groups will not need to adopt and build the app for each new study.

### Functionality

#### Fast Healthcare Interoperability Resources and EHR Integration

SHAPE supports the use of the Health Level 7 (HL7®) Fast Healthcare Interoperability Resources (FHIR®) standard in its data export functionality. FHIR® is a leading innovative standard in the health care field, as it facilitates easier exchange and use of data for research by providing a common way of defining and representing exchangeable content (ie, resources) and a common set of metadata. SHAPE’s data exports are JSON files. Administrators can download the JSON data export in two formats: (1) typical JSON following no data standard or (2) JSON with data mapped to HL7® FHIR® data standards.

SHAPE has been integrated with a third-party application, 1upHealth, to provide participants with the ability to approve a 1-time data pull of their EHRs into the study. This is an innovative feature for SHAPE as participants’ EHRs are not readily accessible. SHAPE connects to 1upHealth’s platform and directs participants to sign into their medical providers’ EHR portals. Once logged in, the participant is provided with the ability to consent to download their health records in FHIR®-formatted files and initiate a 1-time data pull of that information into SHAPE. This ultimately gives the study administrator access to the information.

The benefits of this feature are high for both the participants and study administrators. Researchers can use EHR data to expand data capture and improve the identification of medical conditions, symptoms, current medications, past treatment history, and so on. The addition of EHR data can also minimize missing data and potential recall bias from self-reported medical history data.

From the perspective of the study participants, they have increased transparency into their health records. This occurs through the provision of a 1-time readable version of EHR data that their provider has in their database at the time of import. As a result, they can view their health records in conjunction with their own reported data to facilitate informed conversations with their clinician or clinicians on medical plans. This feature supports the increased importance of exchanging information between health care providers, their patients, and the regulators tasked with ensuring the safety and effectiveness of medications, vaccines, and therapies. The 2 data sources, patient-submitted data and their EHR data, complement each other for a more comprehensive picture of a patient’s medical needs and care.

#### Study Design and Development

This section and the sections that follow include screenshots from a live instance of SHAPE. The screenshots include hypothetical questions developed to demonstrate how administrators can use SHAPE to gather data from patients. All data displayed in this paper are simulated and are not personally identifiable.

For a study to meet institutional review board requirements and properly protect its participants, study administrators must obtain informed consent from each participant. SHAPE provides for efficient collection of these requirements. The SHAPE participant app will only let participants join a study after they have reviewed the terms listed for the study and provided their informed consent within the app. Once the participant has viewed and agreed to the terms, SHAPE delivers a copy of the informed consent to their provided email address and then displays the study to them. SHAPE also tracks the consent in the back-end database for the study’s own audit purposes.

Currently, SHAPE is a secure platform that only invited study participants can access. The administrator shares a unique code with each participant outside the app. The participant then uses the provided code to register with the SHAPE participant app. Once the study is developed and deployed, the participant can access it via the SHAPE participant app, consent to the study, and complete any questionnaires or self-reported events related to their disease throughout the study’s time frame. During the study, the administrator can communicate with participants through in-app messages, emails, and SMS text messaging. If enabled by device owners, the SHAPE participant app can also connect to the device’s native notification center and deliver push notifications to users.

SHAPE supports many of the main modality questions leveraged for studies, such as single-line text, radio button, date and time, checkbox, dropdown, slider, text area, range (limits the minimum and maximum integers a respondent can enter), and information only (there is no response field for the participants; instead, administrators can display images, tables, and text for information-sharing purposes).

When developing questions, study administrators can use complex skip logic rules to ensure that their questionnaire captures all relevant data while only displaying questions applicable to the individual participant. SHAPE supports skip logic rules that can be developed according to the participant’s gender, age, or response to the posed question.

The SHAPE administrator application can also provide administrators with a preview of questionnaires in development. This functionality enables administrators to test the questionnaire’s skip logic while they are building it to ensure that it flows correctly for the participants.

**Figure 2:**
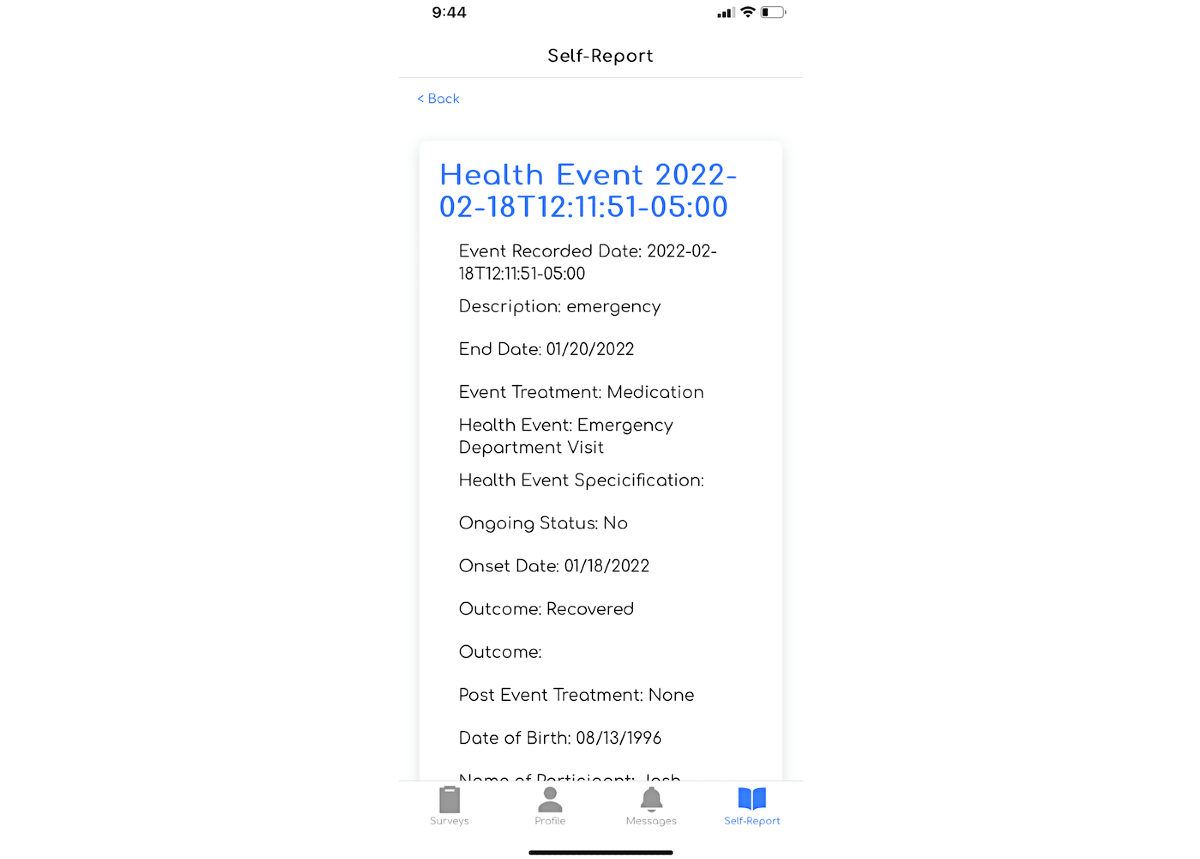
Example self-report in the Survey of Health and Patient Experience app for a health event.

Study administrators can target SHAPE’s self-report functionality to capture patient data on potential health events, results of a clinical visit, or requests to withdraw from the study. The participant can open and submit an entry at their own convenience, in addition to the standard questionnaires deployed within a study. Once a self-report diary entry is submitted, SHAPE holds a receipt of the data collected and displays the information provided to the participant in their diary history view (Figure 2).

## Results

### Use Scenario

**Figure 3:**
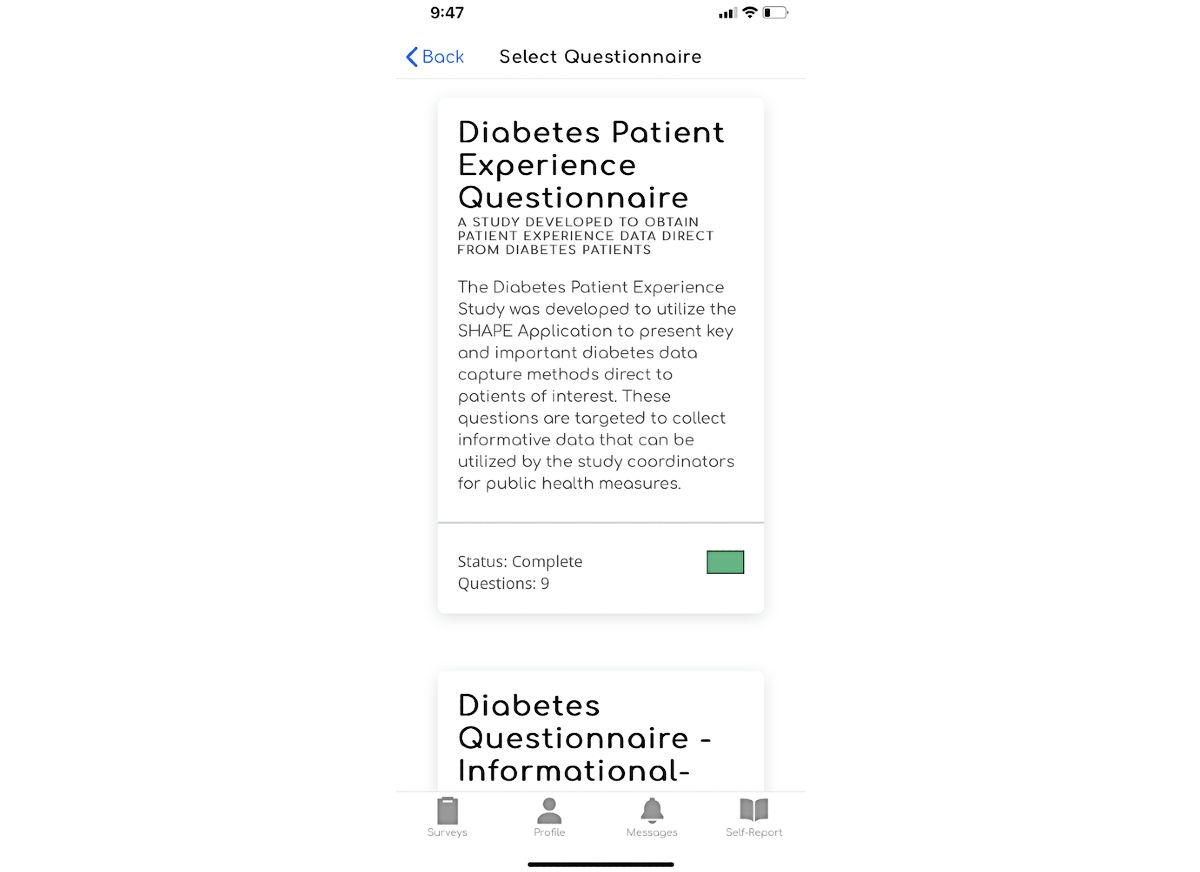
Questionnaire view in the Survey of Health and Patient Experience participant app.

To illustrate how study coordinators can use SHAPE to plan and design patient experience studies, we developed a hypothetical type 1 diabetes cohort study. By using SHAPE, a study coordinator can develop a cohort population based on patients who have signed up for the study. SHAPE can support a study that focuses on obtaining treatment and clinical end points across a series of patient groups while also obtaining longitudinal data through the deployment of a series of questionnaires (Figure 3).

In our hypothetical study, we were interested in understanding treatment patterns and outcomes of patients with diabetes. In this scenario, the study coordinator can develop questionnaires that gather baseline information and health data, followed by a series that captures key treatment end points at the 3-, 6-, and 12-month time points of the study.

**Figure 4:**
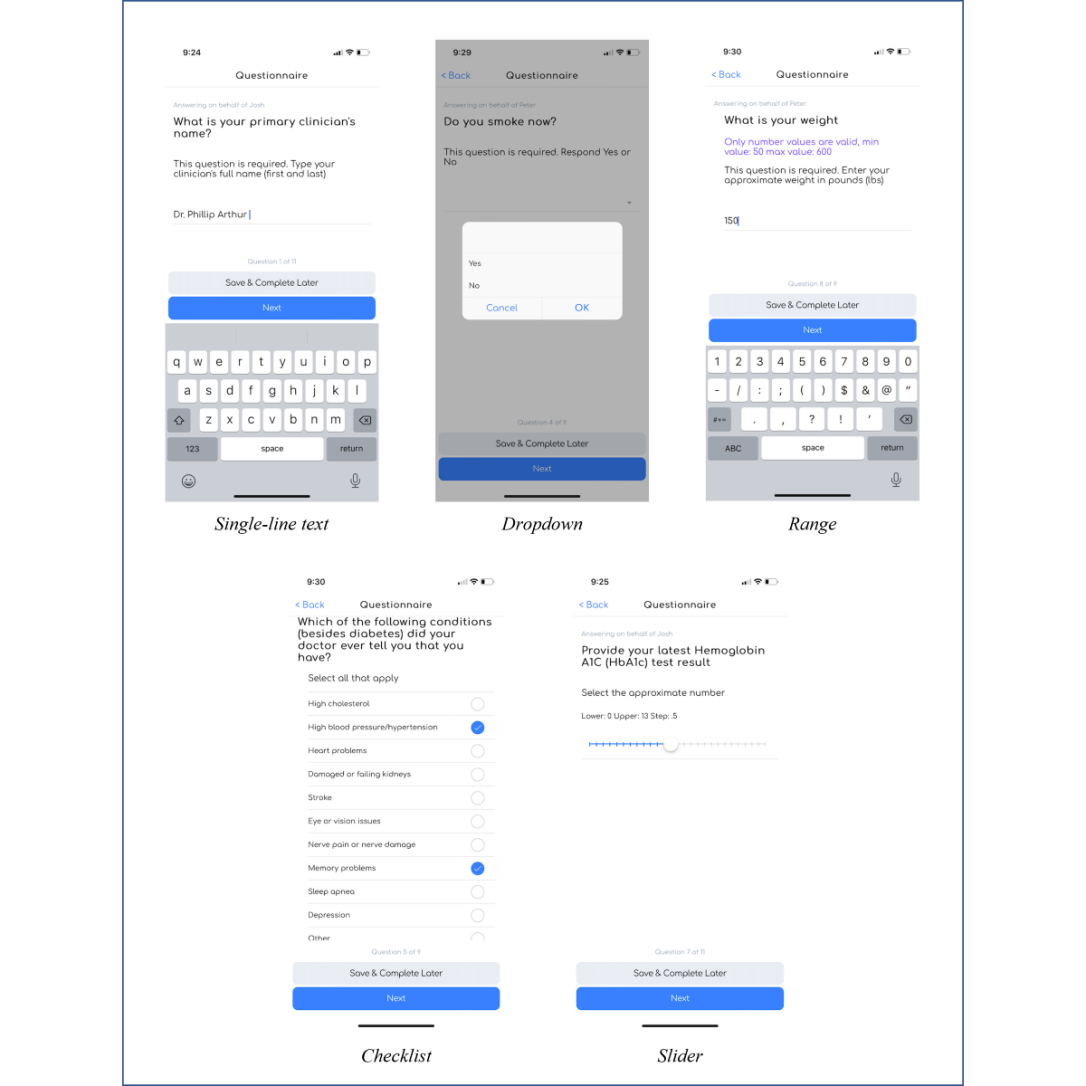
Questionnaire view in the Survey of Health and Patient Experience app question types.

For example, the baseline survey may collect key health and treatment information, such as the names of participants’ primary physicians or specialists, when they were diagnosed with type 1 diabetes, their hemoglobin A_1c_ levels, and other comorbidities. Figure 4 illustrates a selection of these supported baseline SHAPE question types.

**Figure 5:**
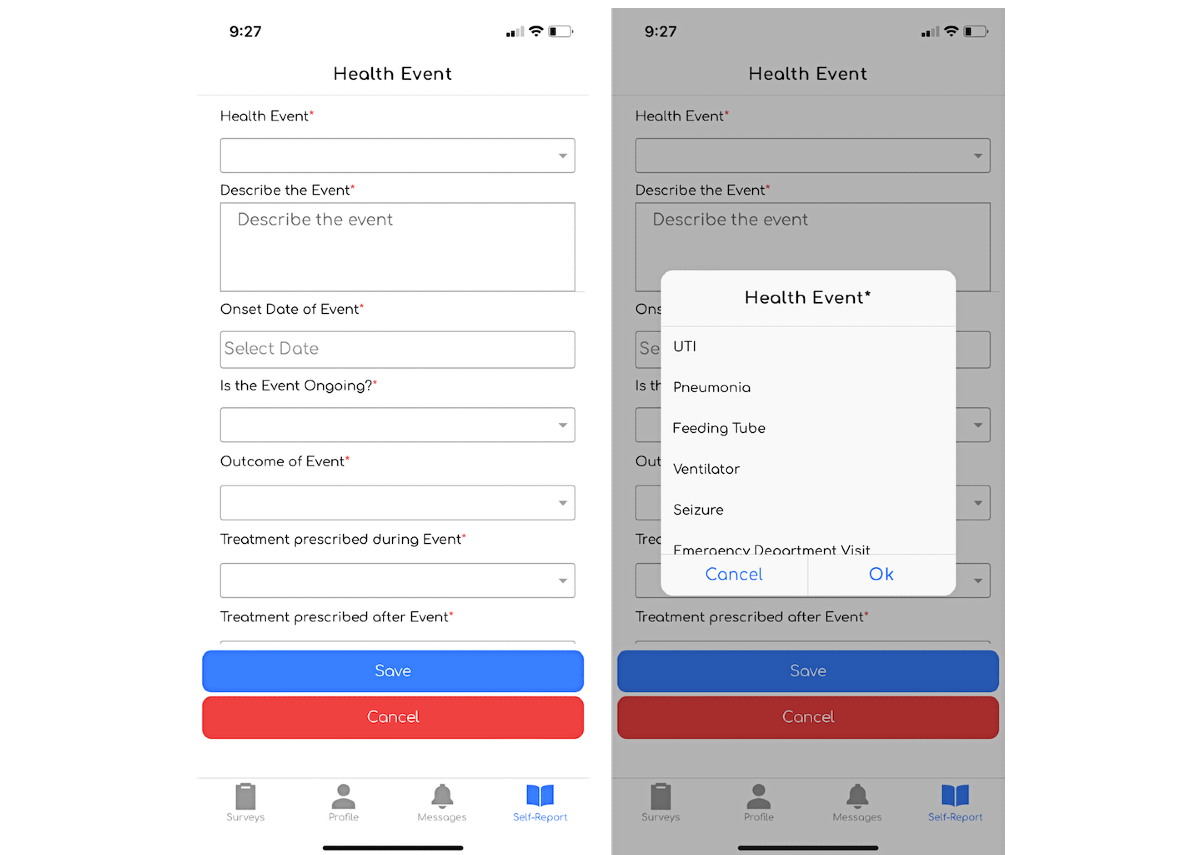
Health event self-report screenshots.

Study coordinators can then create a series of health event diary forms that participants can submit at their leisure. These can capture any health event updates or other key ongoing clinical end points of interest (Figure 5).

**Figure 6:**
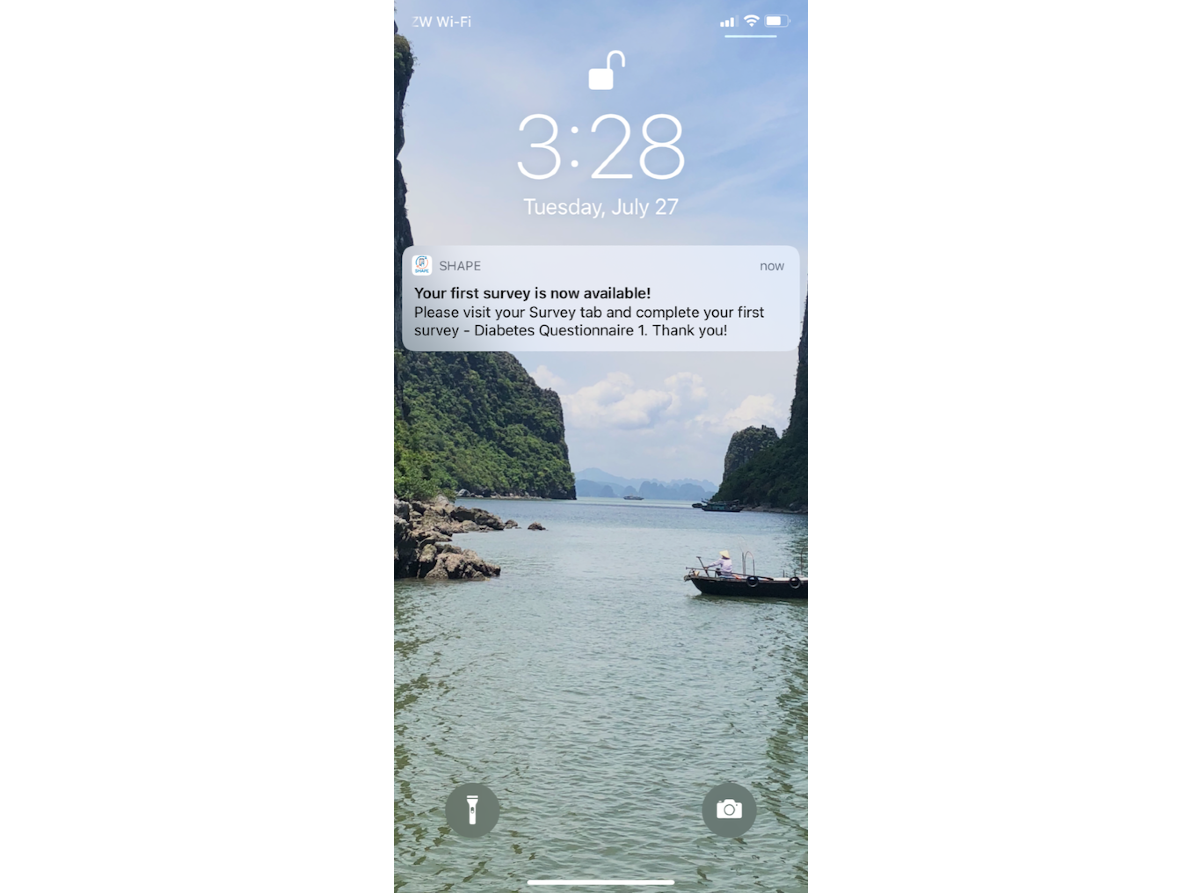
Example push notification on a mobile device.

When a questionnaire is ready to be deployed, the study coordinator has the option to send a push notification (Figure 6) to all mobile app users who have installed the SHAPE participant app and signed up for the study. The coordinator can also develop a message to send via email, SMS text messaging, or the in-app messaging system to inform the study participants of the task.

**Figure 7:**
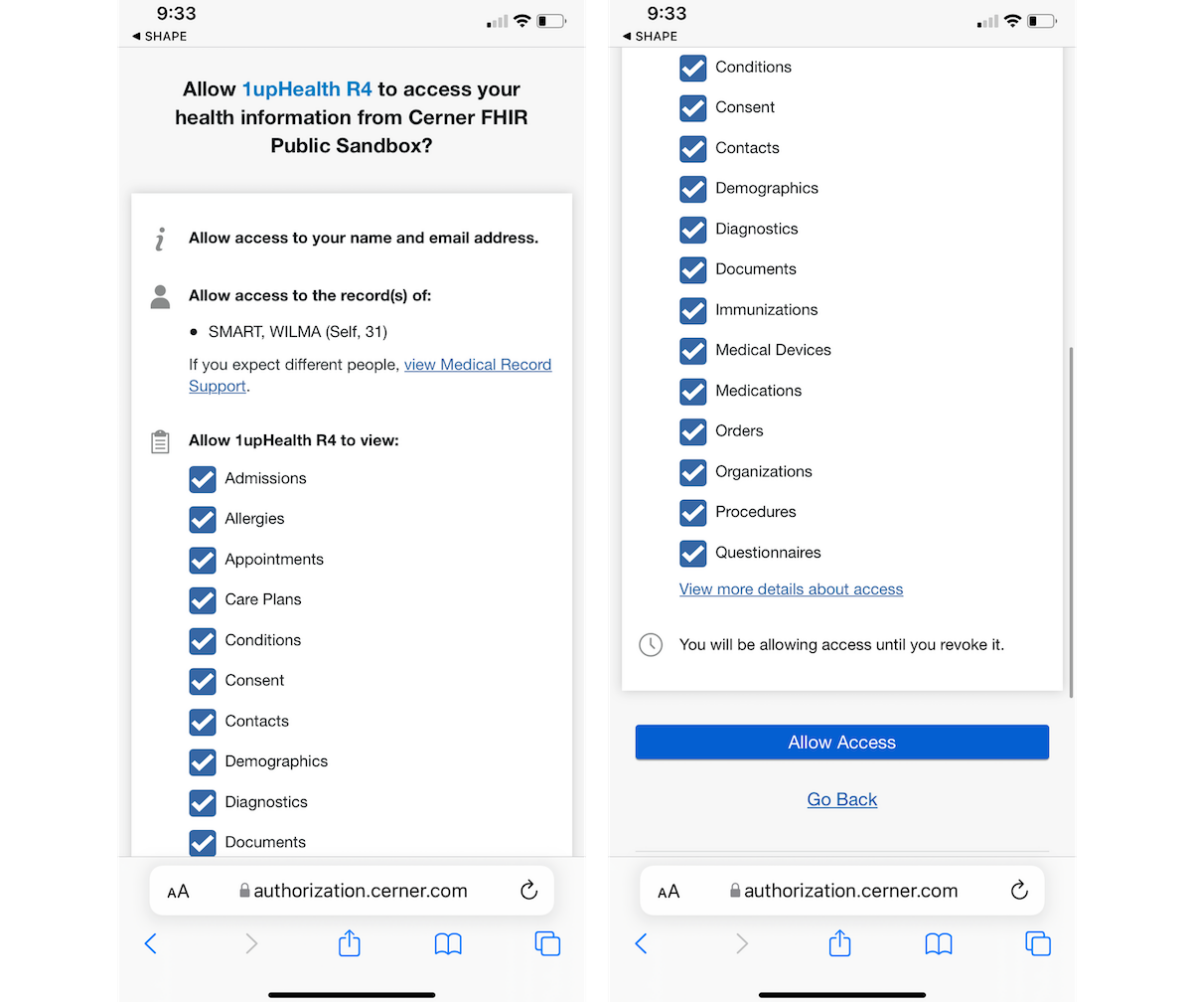
Process for allowing links to the electronic health record.

Study coordinators can deliver a message inviting participants interested in linking their EHRs via the SHAPE participant app to share additional data with the study coordinators (Figure 7). They can then use these data to confirm a patient’s diabetes diagnosis and note any additional information of interest, such as other conditions, medications, or procedures. Access to EHR data allows study coordinators to validate the information from patients’ self-reported health events, addressing potential issues with patient recall by not needing to rely solely on self-reported data.

**Figure 8:**
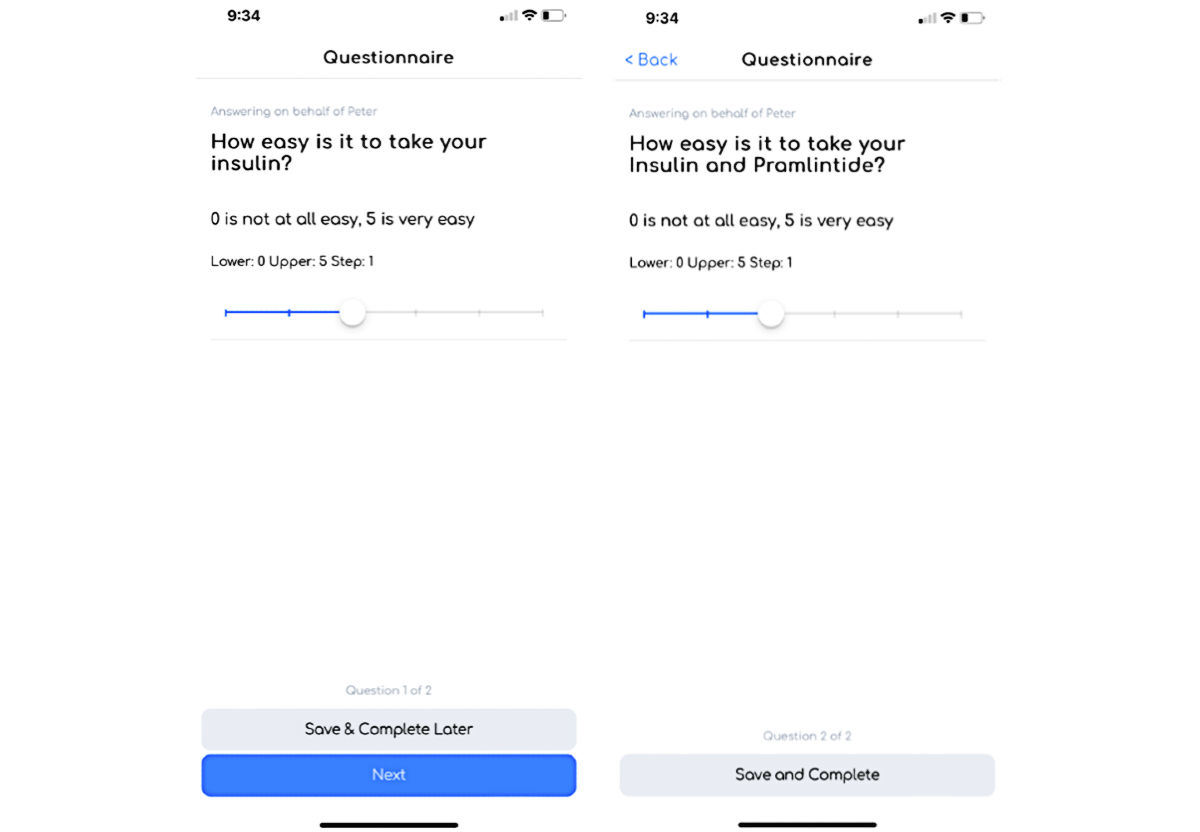
Treatment-specific question examples that capture the use, accessibility, and burden.

**Figure 9:**
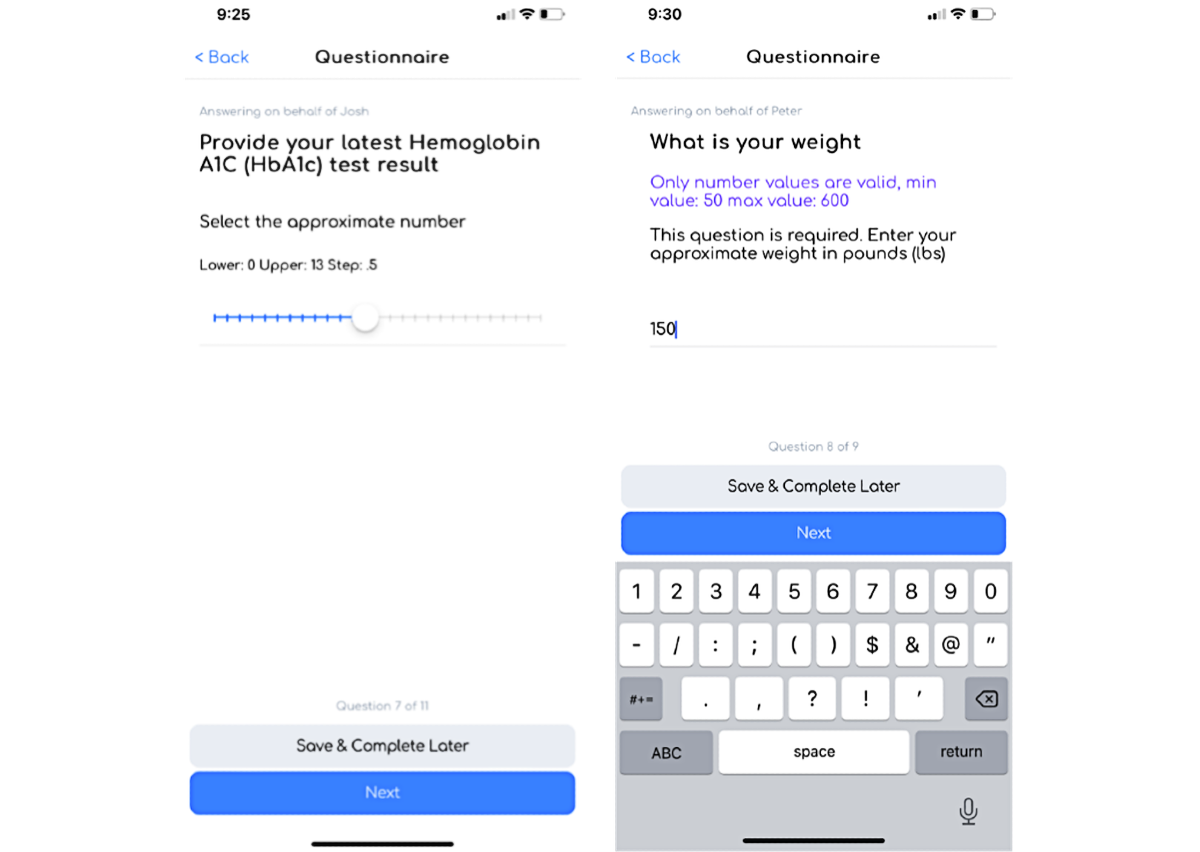
Treatment-specific question examples that capture key vital measurements.

Following baseline questionnaire data collection, the study coordinator is ready to deploy the treatment-specific patient experience questionnaires for longitudinal updates from the 2 treatment populations across the full length of the study. The treatment-specific questionnaires (Figures 8 and 9) can be designed to capture key treatment patient experience end points around use, accessibility, burden, and key vital measurements (eg, hemoglobin A_1c_ level and weight).

**Figure 10:**
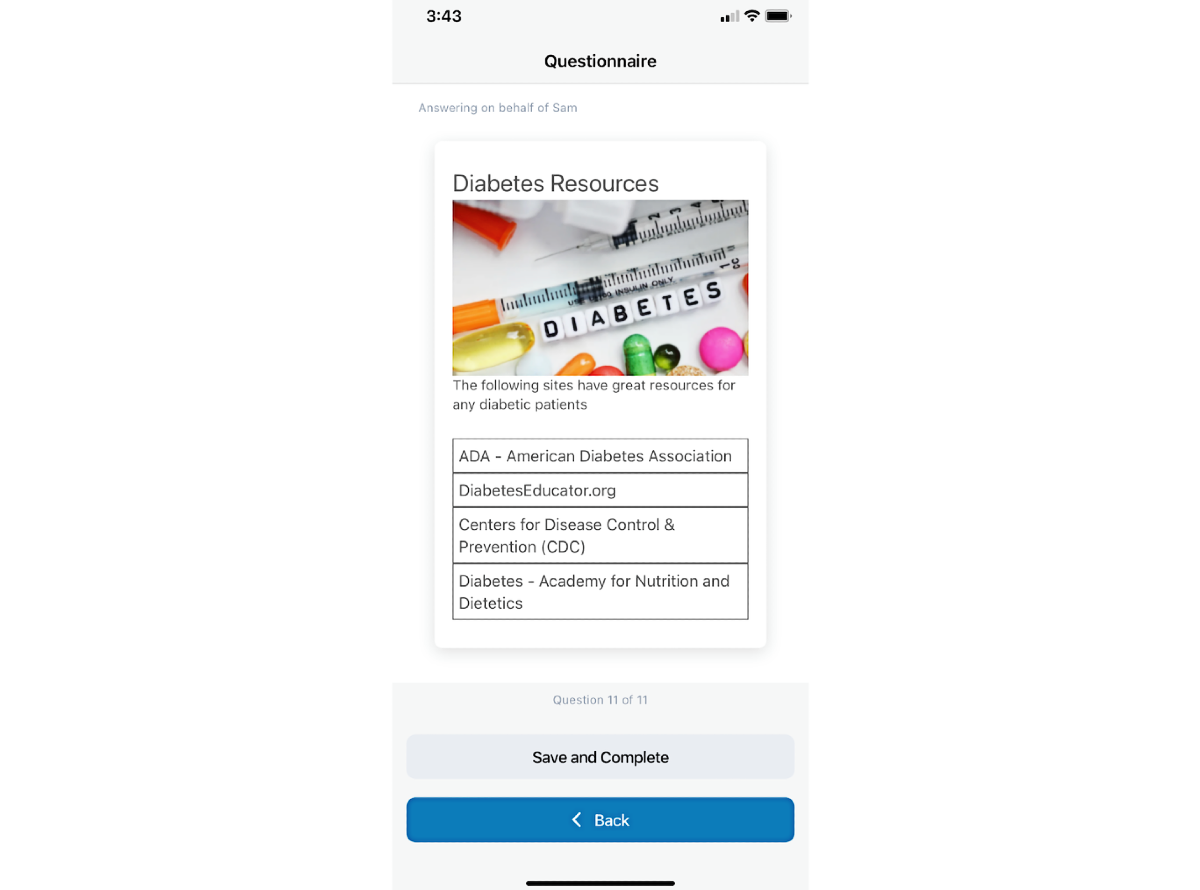
Information only page in the Survey of Health and Patient Experience participant app.

In addition to developing questions for important end point data collection, study coordinators can use the *information only* question type (Figure 10) to share important information or resources with the participants.

SHAPE is an intuitive and comprehensive data-collection tool for a variety of clinical studies. It allows study coordinators to plan, curate, develop, and deploy their questionnaires to obtain important end points directly from patients or through caregivers. The EHR integration functionality enables greater access to the patient’s health records while protecting patient privacy. This allows patients to see their health records at the point of import and provides research coordinators with the use of the data for validation and additional data-capture methods. Overall, this provides a more comprehensive picture of patient experiences with the condition or therapeutic being studied.

### In-Progress Use Case

To address the unmet needs in rare disease therapeutics, the FDA CBER, in collaboration with NORD, launched a pilot Natural History of Metachromatic Leukodystrophy study to implement best practices and processes for designing a regulatory-grade natural history study for rare diseases [25].

This study required a user-friendly app for the deployment of patient experience questionnaires for collecting prespecified study end points. The study is currently in progress in patients with metachromatic leukodystrophy using the SHAPE participant app to provide patient experience data to NORD study coordinators [26,27].

## Discussion

### Principal Findings

As part of the FDA’s mission to facilitate data collection and understanding of patient experiences, SHAPE offers easy-to-use and intuitive SHAPE administrator application and participant app that are appropriate for a variety of studies. In its pilot deployment, SHAPE was used to collect data relevant to regulatory decision-making and patient health outcomes. It aimed to address common challenges of attrition and missing data in natural history studies. The platform allows participants to enter important end point data and other episodic health events at any point during the study from any place with internet access. They can use a computer at their home or submit data on the go with their mobile devices through the SHAPE participant app.

### Strengths

SHAPE has several strengths. First, to use SHAPE, study coordinators are not required to have technical expertise in software development or programming to build, deploy, or collect information from the platform. A study coordinator can log into the SHAPE administrator application and within its 1 interface be able to create studies, incorporate the study’s informed consent, upload participant IDs, curate questionnaires, send communications, view data, export data, and actively open and close participant studies. This offers a low barrier of entry for research teams. SHAPE was designed with the user experience in mind, making it an accessible, flexible, and scalable data-collection platform for both research participants and study coordinators.

In addition, SHAPE allows for easy scalability through its multitenancy ability, which allows for multiple organizations to use SHAPE with their own secure log-ins. This ensures that their studies are secure and restricts access for accounts based on their organization, enabling users to only view and export their organization’s studies and participant data. This feature permits multiple groups within an organization to leverage SHAPE without requiring the development of their own instance of the platform. This leads to substantial cost and time savings.

Finally, SHAPE can potentially reduce the burdens patients and their caregivers may face while participating in conventional regulatory-grade studies at remote clinical sites, such as travel, costs, time, and overall fatigue. It facilitates an easier exchange and use of data for patient-centered research. For example, the EHR integration function allows research participants to explicitly consent to share their EHR data in a deidentified manner, making it a more convenient procedure for data capture while improving the accuracy of collected data and minimizing missing data.

### Limitations

A limitation of SHAPE is that it requires individuals to have access to the internet or smartphones or tablets. It may not be suitable for individuals who are uncomfortable or unfamiliar with using technology. Currently, SHAPE is only available in the English language and lacks accessibility features for individuals who have impaired vision or dexterity (eg, people with Parkinson disease). Future enhancements of the platform could address some of these limitations.

### Comparison With Prior Work

There are several open-source mobile data-collection platforms that support data management for clinical research [11-20]. Many of these serve as important functions and played a significant role in the design and development of SHAPE in terms of its capabilities, functionality, and application. However, few platforms have adequately addressed informed consent processes, such as data security and traceability, that research studies intended for regulatory use require [28]. An exception is the FDA MyStudies app, which was created to conform to stringent federal data privacy and security standards.

SHAPE builds on the prior capabilities of the MyStudies app and includes additional important features and applications, such as customizable questionnaires, self-report, EHR integration capabilities, communication channels, and compatibility with multiple operating systems. These enhancements provide greater ease of use for study coordinators and participants. SHAPE is also unique in its ability to support the use of FHIR® and seamlessly integrate with EHR systems. These capabilities make SHAPE a scalable and flexible data-collection platform that research teams can use to deploy multiple studies without requiring additional configuration by software developers.

Finally, the initial version of the SHAPE platform had good functionalities to support small study groups such as those connected to rare disease research but lacked the automation of studies to support larger cohorts. As SHAPE continues to develop, it will gain broader applicability for research for prevalent diseases. Further automation will be able to support larger study cohorts while keeping costs for study administrators low and study tools easily accessible for study participants. Although rare disease research served as a vital gateway to the development of SHAPE, the platform’s utility can easily expand from rare disease studies to regulatory-grade research for a broad range of diseases.

### Quality Improvement and Future Plans for Enhancement

The SHAPE team has developed guides and training documentation on how to use the SHAPE participant app, SHAPE administrator application, and SHAPE API. The team adds updates to these resources regularly to clarify elements based on user feedback and to highlight upgrades to the tool.

As part of the app’s ongoing quality improvement, the SHAPE team will continue to engage all relevant business and user stakeholders to discuss and prioritize updates. On the basis of the feedback and initial use of the platform, the team has already determined several plans for enhancement.

Future feature development for SHAPE is targeted at increasing the customizability and usability of the tool for the administrators to reduce the study administration burden. Updates will also continually advance SHAPE’s ability to integrate into the native functionalities of a smartphone mobile device, including the camera, calendar, and file records. In addition, future enhancements will further strengthen the platform’s communication functionality and ability to schedule reminders for questionnaires and self-report submissions over set periods, such as every 3 months. This will support longitudinal data capture. There are also plans to provide study administrators with the ability to add respondents to a secondary questionnaire based on a provided response. In addition, the team plans to expand participant registration beyond invites only, allowing app users to find and register for relevant public studies on their own.

Finally, the SHAPE team would like to improve the EHR feature to populate the participant’s consented data collection into a display that is easy to read and that participants can export and print for record-keeping purposes. Future iterations of SHAPE will also position it as an information-sharing channel with a repository of resources that can support clinical trials, study participants, and so on.

### Conclusions

SHAPE is a flexible and scalable app that allows for efficient deployment of regulatory-grade studies, supports multitenancy features, streamlines access to EHR data, eliminates the need for ongoing software developer support, and so on.

This intuitive and comprehensive data-collection platform can be used to support traditional data-capture methods from clinical trials, pragmatic trials, observational studies, and disease registries. To increase the ease of study participation and collect patient experience information, the FDA CBER used SHAPE for a pilot natural history study for a rare disease.

Further customization and automation of this versatile and scalable app will allow for multiple use cases, including larger cohort studies for common diseases. Study administrators can use SHAPE to capture patient perspectives and important clinical data. This provides a more holistic view of each patient, facilitating the goal of achieving better patient outcomes through drug development and delivery of care.

## Appendix

Multimedia Appendix 1 Literature review search strategies.

## Abbreviations

API: application programming interface
CBER: Center for Biologics Evaluation and Research
EHR: electronic health record
FDA: Food and Drug Administration
FHIR®: Fast Healthcare Interoperability Resources
NORD: National Organization for Rare Disorders
REDCap: Research Electronic Data Capture
SHAPE: Survey of Health and Patient Experience
