# Supplementary material for: A Versatile and Scalable Platform That Streamlines Data Collection for Patient-Centered Studies: Usability and Feasibility Study
Source: JMIR Form Res. 2022 Sep 14;6(9):e38579. doi: 10.2196/38579 (PMC9520400; doi:10.2196/38579)
Supplement: Multimedia Appendix 1 [file formative_v6i9e38579_app1.docx]

## Multimedia Appendix 1

### Literature Review Search Strategy 1

| **Concept #** | **Concept** | **Details** | **Citations** |
| --- | --- | --- | --- |
| 1 | Mobile Application OR Mobile App | "mobile applications"[MeSH Terms] OR ("mobile"[All Fields] AND "applications"[All Fields]) OR "mobile applications"[All Fields] OR ("mobile"[All Fields] AND "application"[All Fields]) OR "mobile application"[All Fields] OR ("mobile applications"[MeSH Terms] OR ("mobile"[All Fields] AND "applications"[All Fields]) OR "mobile applications"[All Fields] OR ("mobile"[All Fields] AND "app"[All Fields]) OR "mobile app"[All Fields]) | 28,991 |
| 2 | Data Collection Application OR Data Collection App | (("data collection"[MeSH Terms] OR ("data"[All Fields] AND "collection"[All Fields]) OR "data collection"[All Fields]) AND ("applicabilities"[All Fields] OR "applicability"[All Fields] OR "application"[All Fields] OR "applications"[All Fields] OR "applicative"[All Fields])) OR (("data collection"[MeSH Terms] OR ("data"[All Fields] AND "collection"[All Fields]) OR "data collection"[All Fields]) AND ("australas plant pathol"[Journal] OR "app"[All Fields])) | 79,125 |
| 3 | 1 OR 2 | "mobile applications"[MeSH Terms] OR ("mobile"[All Fields] AND "applications"[All Fields]) OR "mobile applications"[All Fields] OR ("mobile"[All Fields] AND "application"[All Fields]) OR "mobile application"[All Fields] OR ("mobile applications"[MeSH Terms] OR ("mobile"[All Fields] AND "applications"[All Fields]) OR "mobile applications"[All Fields] OR ("mobile"[All Fields] AND "app"[All Fields]) OR "mobile app"[All Fields]) OR ((("data collection"[MeSH Terms] OR ("data"[All Fields] AND "collection"[All Fields]) OR "data collection"[All Fields]) AND ("applicabilities"[All Fields] OR "applicability"[All Fields] OR "application"[All Fields] OR "applications"[All Fields] OR "applicative"[All Fields])) OR (("data collection"[MeSH Terms] OR ("data"[All Fields] AND "collection"[All Fields]) OR "data collection"[All Fields]) AND ("australas plant pathol"[Journal] OR "app"[All Fields]))) | 103,463 |
| 4 | Patient Reported Outcomes | "patient reported outcome measures"[MeSH Terms] OR ("patient"[All Fields] AND "reported"[All Fields] AND "outcome"[All Fields] AND "measures"[All Fields]) OR "patient reported outcome measures"[All Fields] OR ("patient"[All Fields] AND "reported"[All Fields] AND "outcomes"[All Fields]) OR "patient reported outcomes"[All Fields] | 105,512 |
| 5 | Patient Reported Outcome Measures | "patient reported outcome measures"[MeSH Terms] OR ("patient"[All Fields] AND "reported"[All Fields] AND "outcome"[All Fields] AND "measures"[All Fields]) OR "patient reported outcome measures"[All Fields] | 36,223 |
| 6 | 5 OR 6 | "patient reported outcome measures"[MeSH Terms] OR ("patient"[All Fields] AND "reported"[All Fields] AND "outcome"[All Fields] AND "measures"[All Fields]) OR "patient reported outcome measures"[All Fields] OR ("patient"[All Fields] AND "reported"[All Fields] AND "outcomes"[All Fields]) OR "patient reported outcomes"[All Fields] OR ("patient reported outcome measures"[MeSH Terms] OR ("patient"[All Fields] AND "reported"[All Fields] AND "outcome"[All Fields] AND "measures"[All Fields]) OR "patient reported outcome measures"[All Fields]) | 105,512 |
| 7 | 3 AND 6 | ("mobile applications"[MeSH Terms] OR ("mobile"[All Fields] AND "applications"[All Fields]) OR "mobile applications"[All Fields] OR ("mobile"[All Fields] AND "application"[All Fields]) OR "mobile application"[All Fields] OR ("mobile applications"[MeSH Terms] OR ("mobile"[All Fields] AND "applications"[All Fields]) OR "mobile applications"[All Fields] OR ("mobile"[All Fields] AND "app"[All Fields]) OR "mobile app"[All Fields]) OR ((("data collection"[MeSH Terms] OR ("data"[All Fields] AND "collection"[All Fields]) OR "data collection"[All Fields]) AND ("applicabilities"[All Fields] OR "applicability"[All Fields] OR "application"[All Fields] OR "applications"[All Fields] OR "applicative"[All Fields])) OR (("data collection"[MeSH Terms] OR ("data"[All Fields] AND "collection"[All Fields]) OR "data collection"[All Fields]) AND ("australas plant pathol"[Journal] OR "app"[All Fields])))) AND ("patient reported outcome measures"[MeSH Terms] OR ("patient"[All Fields] AND "reported"[All Fields] AND "outcome"[All Fields] AND "measures"[All Fields]) OR "patient reported outcome measures"[All Fields] OR ("patient"[All Fields] AND "reported"[All Fields] AND "outcomes"[All Fields]) OR "patient reported outcomes"[All Fields] OR ("patient reported outcome measures"[MeSH Terms] OR ("patient"[All Fields] AND "reported"[All Fields] AND "outcome"[All Fields] AND "measures"[All Fields]) OR "patient reported outcome measures"[All Fields])) | 2,104 |
| 8 | Humans | NOT other animals (filter) | 2,096 |
| 9 | Language | English (filter) | 2,064 |
| 10 | Study type | NOT (casereports[Filter] OR comment[Filter] OR editorial[Filter] OR letter[Filter] | **2,030** |

### Literature Review Search Strategy 2

| **Concept #** | **Concept** | **Details** | **Citations** |
| --- | --- | --- | --- |
| 1 | ResearchKit | "ResearchKit"[All Fields] | 45 |
| 2 | ResearchStack | "ResearchStack"[All Fields] | 6 |
| 3 | 1 OR 2 | "ResearchKit"[All Fields] OR "ResearchStack"[All Fields] | 46 |
| 4 | Humans | NOT other animals (filter) | 46 |
| 5 | Language | English (filter) | **46** |
